# 

**Published:** 1906-10-13

**Authors:** 


### BOOKS RECEIVED.

W. H. Bailey and Son.

"Infants' Weight Chart for First Year of Life." By Captain Blackham.

William Blackwood and Sons.

"The C.H.Cl$_3$ Problem." 2 vols. By Richard Gill, M.B.

Adlard and Son.

"The Puerperium." By C. Nepean Longridge, M.D.

G. Gill and Sons, Limited.

"Nursing at Home." By J. D. E. Mortimer, M.B., and R. J. Collie, M.D.

Sherratt and Hughes, Manchester.

"Text-book on Diseases of the Heart." By G. Steell, M.D.

T. Fisher Unwin.

"How to Buy a Business." By A. W. Bromley.

Aberdeen University.

"Handbook to City and University of Aberdeen."

### CHARING CROSS HOSPITAL MEDICAL SCHOOL.

The following entrance scholarships have been awarded: The Epsom Scholarship (115 guineas) to Mr. B. F. Eminson. The Livingstone Scholarship (100 guineas) to Mr. N. C. Lake. The Huxley Scholarship (55 guineas) to Mr. H. Smith. Entrance Scholarships have also been awarded to Mr. R. F. Eminson (60 guineas), to Mr. A. E. Huxtable (40 guineas), to Mr. M. Barker (30 guineas). Universities' Exhibitions of 36 guineas each have been awarded to Mr. F. C. Davies (Cambridge University), and Mr. E. A. Ramsden (Oxford University).

### NOTICE TO CORRESPONDENTS.

All MSS., Letters, Books for Review, and other matters intended for the Editor should be addressed The Editor, "The Hospital," The Hospital Buildings, 28 & 29 Southampton Street, Strand, W.C.

The Editor cannot undertake to return rejected MS., even when accompanied by stamped directed envelope.

### Notice to Advertisers and Subscribers.

All Advertisements, Orders for Copies of The Hospital, and Business Communications should be addressed to **THE MANAGER (Not to the Editor),** The Hospital, 28 & 29 Southampton Street, Strand London, W.C.

The Cost of Subscription, post free, is as follows:—Per week, 3½d.; per quarter, 3s. 3d.; per half-year, 6s. 6d.; per annum, 13s. Foreign and Colonial:—Per annum, 19s. 6d., payable, in all cases, in advance.

**NOTICE.—Vol. XL. of "The Hospital" (April to September 1906), in handsome cloth binding, will shortly be on sale at the office of this Journal, price 10s. 6d. Cases for binding the Numbers contained in this Volume 2s. Index to Vol. XL., 3½d., post free.**

**The Monthly Part for October is now ready. Price 1s., by post, 1s. 3d.**

### SHILLING NURSING HANDBOOKS

**POST FREE, 1/1.**

**HOW TO BECOME A CERTIFIED MIDWIFE.**
By E. L. C. APPEL, M.B., B.S., B.Sc.Lond.

**THE NURSING OF SICK CHILDREN.**
By Dr. BURNET.

**SIMPLE INTRODUCTORY LESSONS in MIDWIFERY**
By M. LOANE.

**THE MODERN NURSING OF CONSUMPTION.**
By Dr. JANE H. WALKER.

**THE EXAMINATION OF THE URINE.**
By J. K. WATSON, M.D., M.B., C.M., Author of "A Handbook for Nurses."

**SIMPLE SANITATION:**
The Practical Application of the Laws of Health to Small Dwellings.
By M. LOANE. With an Introductory Chapter on Sanitary Legislation by Dr. A. MEARNS FRASER, Medical Officer of Health, Portsmouth.

**PRACTICAL HINTS ON DISTRICT NURSING.**
By Miss AMY HUGHES.

**FEVERS & INFECTIOUS DISEASES:**
Their Nursing and Practical Management.
By WILLIAM HARDING, M.D.Ed., M.R.C.P.Lond., Author of "Mental Nursing," &c.

**NOTES ON PHARMACY & DISPENSING for NURSES.**
New and Revised Edition.
By C. J. S. THOMPSON.

**SICK NURSING AT HOME.**
By Miss L. G. MOBERLEY.

**SYLLABUS OF LECTURES TO NURSES.**

By ANDREW DAVIDSON, M.D.

### The Scientific Press, Ltd.

**28 & 29 Southampton Street, Strand, London, W.C.**

PUBLISHERS OF NURSING TEXT-BOOKS, CHARTS, AND CASE-BOOKS OF ALL DESCRIPTIONS.

*Catalogue of Nursing Manuals sent post free on application*

### You have often Needed a Book

That would aid you in an emergency.

That would help you to remember some little point you had forgotten or about which you had a doubt.

That would prove of assistance in difficulty.

That would convey information clearly and concisely on Diseases; their Symptoms and Nursing Treatment.

### The Nurse's Enquire Within

**is a Pocket Encyclopædia of valuable nursing information alphabetically arranged, and**

### Will Fill a Want that has long been felt by Nurses everywhere. It is bound in limp cloth boards with rounded corners, and its size and weight will admit of its being carried conveniently in the apron pocket without injury thereto.

### The Nurse's Enquire Within will be published shortly at the price of **2/-** net, by post **2/3.**

The demand for this book will undoubtedly be very great, Nurses are therefore strongly advised to order copies without delay to avoid disappointment, as the edition will be limited.

### The Scientific Press, Limited.

*NURSING PUBLISHERS AND BOOKSELLERS,*
28/29 SOUTHAMPTON STREET,
STRAND, LONDON, W.C.

